# Incidental retrieval of emotional contexts in post-traumatic stress disorder and depression: An fMRI study

**DOI:** 10.1016/j.bandc.2008.05.008

**Published:** 2009-02

**Authors:** Matthew G. Whalley, Michael D. Rugg, Adam P.R. Smith, Raymond J. Dolan, Chris R. Brewin

**Affiliations:** a Sub-Department of Clinical Health Psychology, University College London, Gower Street, London WC1E 6BT, UK; b Wellcome Department of Imaging Neuroscience, Institute of Neurology, University College London, UK; c Center for the Neurobiology of Learning and Memory, University of California, Irvine, USA

**Keywords:** PTSD, Memory, Trauma, Imaging

## Abstract

In the present study, we used fMRI to assess patients suffering from post-traumatic stress disorder (PTSD) or depression, and trauma-exposed controls, during an episodic memory retrieval task that included non-trauma-related emotional information. In the study phase of the task neutral pictures were presented in emotional or neutral contexts. Participants were scanned during the test phase, when they were presented with old and new neutral images in a yes/no recognition memory task. fMRI results for the contrast between old and new items revealed activation in a predominantly left-sided network of cortical regions including the left middle temporal, bilateral posterior cingulate, and left prefrontal cortices. Activity common to all three groups when correctly judging pictures encoded in emotional contexts was much more limited. Relative to the control and depressed groups the PTSD group exhibited greater sensitivity to correctly recognised stimuli in the left amygdala/ventral striatum and right occipital cortex, and more specific sensitivity to items encoded in emotional contexts in the right precuneus, left superior frontal gyrus, and bilateral insula. These results are consistent with a substantially intact neural system supporting episodic retrieval in patients suffering from PTSD. Moreover, there was little indication that PTSD is associated with a marked change in the way negatively valenced information, not of personal significance, is processed.

## 1 Introduction

Behavioural studies of memory for emotionally neutral material have indicated that in post-traumatic stress disorder (PTSD) there is a small but consistent deficit that is greater for verbal than non-verbal material (Brewin, Kleiner, Vasterling, & Field, 2007). The most characteristic aspect of memory functioning in PTSD, however, is the involuntary retrieval of distressing memories, usually involving visual images of the traumatic episode. Neuroimaging research on PTSD has in the great majority of cases studied the retrieval of negative memories using a ‘script-driven imagery’ paradigm where individuals with similar trauma exposure who either do or do not suffer from PTSD are read a description of their traumatic experience and are asked to recall the events as vividly as possible. In this study we attempt to compensate for some of the limitations of the script-driven imagery paradigm by investigating the neural correlates of retrieving memories in which the emotional valence is created experimentally rather than through personal experience.

Previous script-driven imagery studies have found that PTSD is associated with reduced activity in the medial prefrontal cortex (mPFC) and anterior cingulate cortex (ACC) (Bremner et al., 1999; Lanius et al., 2001; Shin et al., 1999). In other studies attempts have been made to provoke symptoms by playing combat-related or neutral sounds to veterans with PTSD. These have found increased amygdala activity in the combat condition (Liberzon et al., 1999; Pissiota et al., 2002). Disrupted activity in the mPFC has been associated with a failure to inhibit amygdala activity, leading to a hypothesised failure to extinguish fear responses (Morgan & LeDoux, 1995). However, many of the available neuroimaging studies have been criticised on the grounds that, due to the lack of a clinical control group, results are not necessarily specific to PTSD (Hull, 2002). Other reviews have pointed out that neuroimaging studies have yet to provide evidence relevant to theoretically important manifestations of PTSD, such as intrusive thoughts and memories (Frewen & Lanius, 2006; Liberzon & Martis, 2006).

The interpretation of script-driven imagery studies is complicated by several more specific methodological factors. Firstly, since the neural substrates of emotional processing in general are known to be altered in PTSD (Armony, Corbo, Clément, & Brunet, 2005; Phan, Britton, Taylor, Fig, & Liberzon, 2006; Rauch et al., 2000; Shin et al., 2001), any effects on memory retrieval are potentially confounded with effects associated with differential on-line processing of presented traumatic stimuli. There is now good evidence to suggest that different structures are involved in the processing of internally generated versus externally generated events (Lieberman, 2007; Reiman et al., 1997), and this distinction is blurred by script-driven imagery procedures. Second, without a condition involving negative but non-traumatic material it is unclear whether the observed effects of script-driven imagery are specific to traumatic memories or are more general. It is possible that in PTSD there is a disturbance in the retrieval of emotional memories more generally, or even of neutral memories.

Little is known about the neural correlates of retrieving non-traumatic memories in PTSD. We found only two studies (Bremner et al., 2003; Shin et al., 2004), both of which employed block designs that do not allow determination of whether observed effects relate to processing of individual emotional memories or reflect adoption of a particular cognitive set during an extended period of exposure to old and new emotional information.

The present study is therefore one of the first to investigate emotional (non-traumatic) and neutral episodic memory retrieval in PTSD, while addressing concerns raised in reviews of previous neuroimaging work (Hull, 2002; Liberzon & Martis, 2006). Comparison groups included trauma-exposed controls and depressed patients. Because depression is commonly comorbid with PTSD the use of depressed controls was designed to enable us to draw stronger conclusions about neural activity associated specifically with PTSD. Like PTSD patients, the depressed also frequently experience intrusive memories and tend to be prescribed similar kinds of medication. They do not always experience traumatic events meeting PTSD Criterion A, however, and hence an additional non-clinical trauma control group is appropriate.

In order to isolate the process of retrieving emotional information we employed an incidental memory paradigm in which during a training phase initially neutral stimuli were paired with either neutral or emotional contexts. The test phase consisted of a recognition task in which participants indicated whether a series of neutral objects were or were not familiar. Previous research with this paradigm (Maratos & Rugg, 2001; Maratos Dolan, Morris, Henson, & Rugg, 2001; Smith, Henson, Dolan, & Rugg, 2004; Smith, Henson, Rugg, & Dolan, 2005) has shown that neural activity varies systematically when participants recognise items previously encoded in emotional versus neutral contexts. Encoding neutral items in an emotional context is thought to affect neural activity at retrieval by either changing the value of the neutral item, or by its becoming a cue for the retrieval of contextual information.

Our aim was to investigate whether PTSD was associated with changes in the neural networks involved in the retrieval of episodic memories in general (independent of stimulus valence), as well as in the retrieval of positive and negative versus neutral memories. Based on previous experiments using similar paradigms (see above) it was anticipated that regions involved in emotional retrieval would include those associated with the processing of emotional stimuli, such as the amygdala and cingulate cortex (see Zald, 2003 and Vogt, 2005 for reviews). Because we were using emotional but non-traumatic stimuli we hypothesised that the PTSD group would exhibit a substantially intact episodic retrieval system.

## 2 Methods

### 2.1 Participants

Participants were 48 right-handed individuals without a history of head injury, neurological disorders, or other major medical conditions. Assessed using the SCID (First, Spitzer, Gibbon, & Williams, 1997), sixteen patients met DSM-IV diagnostic criteria for current PTSD (PTSD group). Sixteen participants had experienced traumatic events similar in magnitude to the PTSD group, but had not developed PTSD (trauma-exposed control group). Sixteen patients meeting DSM-IV criteria for current major depression but not PTSD were also tested (depressed group). Patients in the PTSD and control groups had experienced a range of traumas, including involvement in the July 7th 2005 London bombings (PTSD = 4; control = 5), childhood sexual abuse (PTSD = 5; control = 0), rape/sexual assault (PTSD = 1; control = 2), military trauma (PTSD = 3; control = 1), interpersonal violence (PTSD = 3; control = 1). Time since the index trauma ranged from 4 months to 37 years.

Patient demographics and scores on clinical measures are given in Table 1. Groups differed significantly on the Beck Anxiety Inventory (BAI; Beck, Steer, & Brown, 1996), *F*(2, 45) = 40.70, *p* < .001. Scores for the PTSD and Depressed groups differed significantly from the control group on the Beck Depression Inventory (BDI; Beck et al., 1996), *F*(2, 45) = 63.16, *p* < .001. Participants in the PTSD group scored significantly higher than control participants on the Impact of Event Scale-Revised (IES-R; Weiss & Marmar, 1997), *t*(24.8) = 11.53, *p* < .001 (*df* corrected for heterogeneity of variance). Scores for the PTSD group were significantly greater than those of the Control group on all four measures of the Post-traumatic Stress Disorder Scale (PDS, Foa, 1995), *t*(30) = 11.19, 16.51, 11.9, 18.79, *p* < .001 for PDS b, c, d & total scores, respectively.

### 2.2 Stimulus materials and list construction

Stimuli consisted of two picture components, an object superimposed on a background context. Objects were presented within a yellow box to demarcate their separation from backgrounds, which were drawn principally from the International Affective Pictorial System (IAPS: Lang, Bradley, & Cuthbert, 1999), a series of pictures with standardised ratings for valence and arousal. A small number of the background materials consisted of photographs additional to the IAPS set, which were used to replace certain items unsuitable for particular PTSD patients (full image set available from the authors upon request).

Background images used in the study were selected from pilot studies using 10 British subjects (5 female, mean age = 27.5 years, standard deviation 3.3 years). Subjects in this pilot study were shown images from the IAPS set and rated them using separate 5-point Likert scales for valence and arousal. Some images used in the original Smith et al. (2004) study were deemed to be too upsetting for patients, in particular images involving themes of assault, road traffic accidents, or sexual material were removed. For a few pictures, the ratings of these subjects differed significantly from the IAPS norms. These pictures were excluded, as were those which showed across-subject variances in rating scores greater than 0.75.

The selected set of backgrounds consisted of 150 pictures subdivided into three sets that were either negatively (mean 1.56, *SD* = 0.27), neutral (mean 3.04, *SD* = 0.14) or positively (mean 4.07, *SD* = 0.20) valenced as determined by the ratings of the pilot group. Each of the valence categories contained images of people, animals, objects and landscapes.

The objects that were superimposed on these backgrounds came from a wide range of semantic categories (e.g. tools, furniture, clothing, machinery). They were assessed for emotional valence by the same subjects who rated the backgrounds. Objects that deviated from neutral valence (valence mean <2.7 or >3.3; variance >0.75) or were arousing (mean arousal rating >2) were excluded. A total of 400 objects were employed as critical stimuli.

At study, subjects were presented with one of eight study lists. Each list included the 150 selected backgrounds, arranged pseudorandomly, with no more than three pictures from the same valence category presented consecutively. Each critical object was paired with a neutral background in one list, a negative background in another, a positive background in a third, and was available as a new item for the remaining lists. The study phase was separated into two parts to allow rest breaks. Each study list was paired with two test lists, consisting of 150 old items, 125 new items, and 4 filler items. Each test list utilised a different set of new items, and each subject was tested on only one of the two test lists. One hundred null events were randomly interspersed within the test list, allowing estimation of item-evoked responses relative to baseline. A practice study list of six backgrounds/object pairs and a practice test list of nine objects were also constructed and used to train subjects before the experiment proper.

### 2.3 Study procedure

**Fig. 1:**
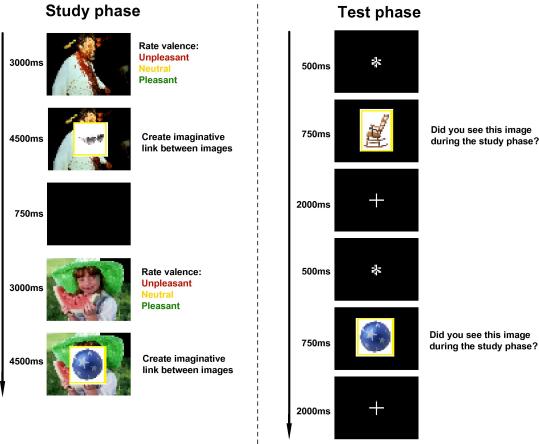
Schematic representation of the study and test phases of the experiment. During the study phase participants rated the valence of background images, and then created an imaginative link between the background and foreground image. During the test phase participants viewed a series of foreground images and were asked to identify items they had seen during the study phase.

All procedures were approved by the National Hospital for Neurology and Neurosurgery & Institute of Neurology Joint Research Ethics Committee. All patients gave written informed consent. Participants completed the study phase of the experiment in a testing room near the scanner. Stimuli were presented via a pc running Matlab (Mathworks, Nathick, MA) and Cogent (http://www.vislab.ucl.ac.uk/Cogent/). Stimuli were in direct view of the participant at a distance of approximately 30 cm. The background was initially presented on the screen for 3 s (Fig. 1). During this time, subjects indicated whether they judged the backgrounds to be pleasant, unpleasant or neutral, using keyboard responses to assign them to these three categories. Three seconds after presentation of the context, the critical object was superimposed centrally upon the background, and subjects were required to imagine a connection between background and object to aid them in a subsequent recall task. The object and background were presented together for 4.5 s, and the screen was then blanked for 750 ms before presentation of the next background. Six practice trials were given, during which the subjects were required to describe verbally the connections they had made between background and object, thereby ensuring they understood the task. During the study phase proper the connections were not verbalised. A rest break was given after 75 trials.

### 2.4 Test procedure

The test phase was conducted inside the scanner, following the study phase with as short a delay as possible. In the test phase stimuli were presented via a mirror mounted on the head coil of the fMRI scanner, in direct view of the supine participant, at a distance of approximately 50 cm from the projection screen. On each trial a white asterisk was presented against a black background for 500 ms, following which the test item was presented for a duration of 750 ms. This was followed by a white fixation cross on a black background for approximately 2 s before presentation of the asterisk denoted the imminent arrival of a new trial. This sequence of events gave a stimulus onset asynchrony (SOA) of 3.25 s. One hundred ‘null events’, consisting of the white fixation cross for an additional 1.25 s in place of the white asterisk and test item, were incorporated into the test list, allowing estimation of baseline. Subjects were instructed to respond, as quickly and accurately as possible, with one button of the keypad when the object depicted had been presented in the preceding study phase, and with another button when it was being viewed for the first time. Assignment of finger responses was counterbalanced across subjects. The test list was split into two equal parts, with the first two stimuli of each subphase being filler items (with their presentation timed to take place during the acquisition of dummy volumes). Before the test phase proper, an example test phase was given, containing the six items from the practice study list, plus three new items. None of the these items appeared in the subsequent test list.

### 2.5 Imaging and image processing

MRI data were acquired from a 1.5-T Siemens SONATA system (Siemens, Erlangen, Germany) equipped with a head receiver coil. Functional images were acquired with a gradient echo-planar T2∗ sequence (TE = 50 ms) using BOLD (blood-oxygenation level dependent) contrast, with a repetition time (TR) = 2.7 s, giving an effective sampling rate of approximately 2 Hz. Thirty slices of 2.5 mm thickness were acquired, with an interslice gap of 1.25 mm, giving nearly whole brain coverage with the exception of the vertex and superior parietal lobe. Data were acquired during two separate sessions, with the first five volumes of each session discarded to allow for T1 equilibration effects. Data were acquired using a sequence optimised to reduce susceptibility artefacts near air/tissue interfaces (Weiskopf, Hutton, Josephs, & Deichmann, 2006). A magnetic (B0) field map image was collected after the second session and was used to unwarp the echo-planar images (Cusack, Brett, & Osswald, 2003). Subjects were placed in a light head restraint within the scanner to limit head movement during acquisition. A T1-weighted structural image was also acquired following the functional acquisition. Image processing and statistical analysis was conducted using Statistical Parametric Mapping software (SPM5; Wellcome Department of Imaging Neuroscience; (http://www.fil.ion.ucl.ac.uk/spm). Functional images were realigned and unwarped using magnetic fieldmaps and slice-time corrected. Each participant’s structural image was coregistered with the mean functional image, then segmented. Parameters from the segmentation were used to normalise the functional images into a standardised space as implemented in SPM5, and were finally smoothed with a Gaussian kernel with full-width half-maximum of 8 mm. Estimated translation and rotation parameters were inspected and never exceeded 3 mm per run.

### 2.6 Statistical analysis of images

Data were analysed in SPM5 using a random effects analysis. Test data were modelled as six discrete event types: new items were separated into those that were correctly rejected as new (correct rejections), or incorrectly judged to be old (false alarms). Old items from each of the three categories of old items (negative, neutral, and positive according to subjective ratings) were separated according to whether they had been judged as old (hits, modelled separately) or incorrectly judged to be new (misses, modelled as one event type independent of the category of the item). Trials where no response was logged, or where the participant pressed both buttons, were logged as misses. Principal contrasts were between those events that received correct responses (i.e. hits and correct rejections). Events were modelled with delta functions convolved with a standard canonical haemodynamic response function. For some contrasts, positive and negative hits were collapsed to form a single ‘emotional hit’ condition.

## 3 Results

### 3.1 Behavioural results

During the study phase participants classified the valence of background images as either positive, neutral, or negative. Table 2 shows the result of this classification broken down by group. In 4 out of 144 cases individuals’ responses were identified as being greater than 2 standard deviations above the group mean and were replaced with scores one unit greater than the next highest score (Tabachnick & Fidell, 1996).

A 3 (Group) × 3 (Background valence) mixed-model ANOVA was conducted to analyse how many background images participants classified as being negative, neutral or positive during the study phase. There was no main effect of group *F*(2, 45) = 1.15, *p* = .324, but there was a significant main effect of valence *F*(1.66, 74.73) = 4.72, *p* = .011 (*df* corrected for heterogeneity of variance), and a significant interaction *F*(3.32, 74.73) = 7.41, *p* < .001 (*df* corrected for heterogeneity of variance). Subsidiary tests showed that the number of items depressed participants classified as neutral was significantly higher than the number of items they classified as negative (*t*(15) = 3.44, *p* = .005) or positive (*t*(15) = 3.31, *p* = .005) (‘neutral bias’). PTSD patients and trauma controls, in contrast, classified similar numbers of items as neutral, negative, and positive.

Table 2 also shows participants’ accuracy in identifying items presented at test. It is important to note that all items presented during the test phase were neutral. For the purposes of this analysis we will refer to neutral items encoded in the differently valenced contexts as positive, neutral, or negative respectively. A 3 (Group) × 4 (Target) mixed model ANOVA was run to test how accurately participants discriminated old items seen in a negative, neutral, or positive context, and new items. Mauchly’s test of sphericity was significant and degrees of freedom were adjusted with a Greenhouse-Geisser procedure. There was a significant main effect of target, *F*(2.31, 103.96) = 27.24, *p* < 0.001, and group, *F*(2, 45) = 6.73, *p* = 0.003, but no significant interaction, *F*(4.62, 103.96) = 2.18, *p* > 0.05 (all df’s corrected for heterogeneity of variance). Analysis of the target effect revealed significant differences in accuracy for identifying new items compared with negative (*t*(82.99) = 5.07, *p* < 0.001), neutral (*t*(86.19) = 5.38, *p* < 0.001), and positive (*t*(83.01) = 4.71, *p* < 0.001) items (Levene’s test indicated unequal variances, degrees of freedom have been adjusted accordingly). Recognition of old items was equally accurate regardless of the valence of the context in which they had been encoded.

Analysis of the group effect revealed significant differences in accuracy between PTSD and Control groups (*t*(112.29) = 3.78, *p* < .001), and between Depressed and Control groups (*t*(126) = 5.76, *p* < .001), but no differences between PTSD and Depressed groups.

### 3.2 fMRI results

Primary aims of the fMRI analysis were to identify regions common to all three groups for principal contrasts of interest, and to identify areas demonstrating increased or reduced activity in the PTSD group relative to the control or depressed groups. Principal contrasts of interest were (i) for the three hit conditions (neutral, negative, positive) vs. correct rejections (old > new effects), (ii) between a collapsed emotional hit condition (weighted combination of positive and negative hits) and neutral hits (valence-independent effects), and (iii) between positive, negative and neutral hits separately (valence-specific effects). The reported contrasts are thresholded at a significance level of *p* < .001 uncorrected with a spatial extent of at least five contiguous voxels. To identify regions where effects of equivalent magnitude were common to all three groups contrasts were computed for all 48 participants, thresholded at *p* < .001 uncorrected, then exclusively masked with cross-group interaction effects (e.g. PTSD vs. depressed) thresholded at *p* < .05 two-tailed (the lower significance here increases the confidence with which it can be concluded that two contrasts did not overlap). To identify regions demonstrating greater activity in the PTSD group than in the depressed or control groups, one-tailed PTSD>depressed and PTSD>control contrasts were inclusively masked, both thresholded at *p* < .005 to produce a conjoint significance of *p* < .001 uncorrected. To identify regions demonstrating less activity in the PTSD group than in the depressed or control groups, one-tailed Depressed>PTSD and Control>PTSD contrasts were inclusively masked, both thresholded at *p* < .005 to produce a conjoint significance of *p* < .001 uncorrected.

#### 3.2.1 Common effects

**Fig. 2:**
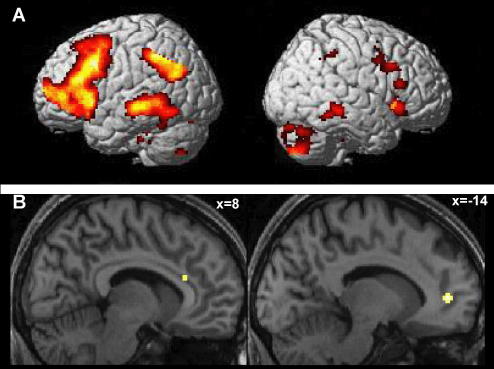
Activity common to all three groups for the (A) old > new, (B) emotional hit > neutral hit contrasts. Projected on to (A) surface representation, (B) representative subject’s anatomy. All contrasts thresholded at *p* < .001 uncorrected.

Effects common to all three groups for the hits > correct rejection contrast revealed significant activations in a broad range of predominantly left-sided regions including left prefrontal cortex, caudate, precuneus, cingulate cortex, middle temporal gyrus, and in the right temporal gyrus, inferior frontal gyrus and middle frontal gyrus, as shown in Fig. 2. These areas are similar to those reported in previous studies of recognition memory (Henson, Rugg, Shallice, Josephs, & Dolan, 1999; Smith et al., 2004; Smith et al., 2005; Yonelinas, Otten, Shaw, & Rugg, 2005; see Rugg, Otten, & Henson, 2002 for review).

Effects common to all three groups for the emotional > neutral contrast were observed in right mid-cingulate cortex and left pregenual anterior cingulate (Fig. 2 and Table 3). This region of left pregenual cingulate was further identified in the contrast of positive and neutral hits common to all groups. Effects common to all three groups for the contrast of negative and neutral hits were observed in the left dorsolateral prefrontal cortex (DLPFC) (Table 3).

**Fig. 3:**
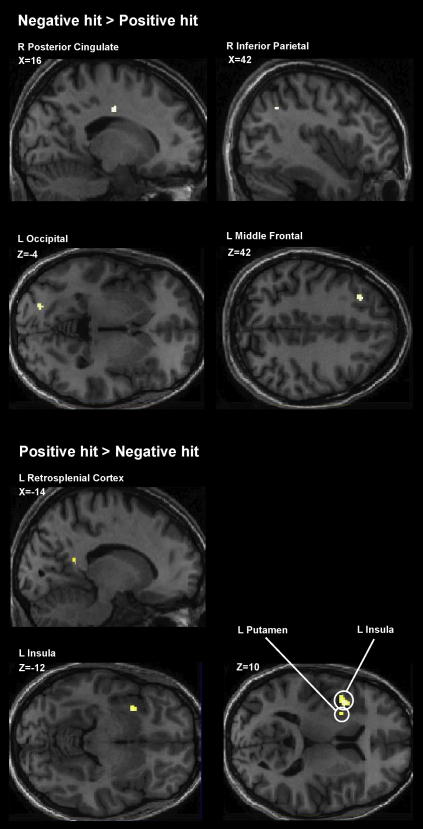
Activity common to all three groups for the negative > positive and positive > negative hit contrasts. Projected on to a representative subject’s anatomy. All contrasts thresholded at *p* < .001 uncorrected.

Common effects for the negative hit > positive hit contrast were observed in the right posterior cingulate, left middle frontal cortex, left occipital cortex and right inferior parietal cortex. Common effects were observed in the left insula, putamen and retrosplenial cortex for the positive hits > negative hit contrast (see Table 3 and Fig. 3).

#### 3.2.2 Altered activity in the PTSD group

**Fig. 4:**
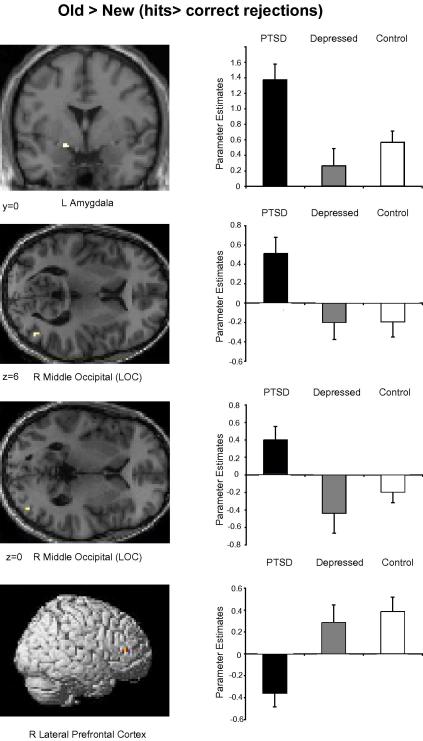
Parameter estimates (arbitrary units) for regions demonstrating additional or decreased activity in the PTSD group relative to the control and depressed groups for the old > new contrast. Statistical parametric maps thresholded at *p* < .001 uncorrected.

For the old > new contrast (hits > correct rejections) the PTSD group demonstrated greater activity than the depressed or control groups in the left dorsal amygdala (close to ventral striatum), and right middle occipital cortex; PTSD participants also demonstrated significantly less activity in a region of the right lateral prefrontal cortex (Table 4 and Fig. 4).

**Fig. 5:**
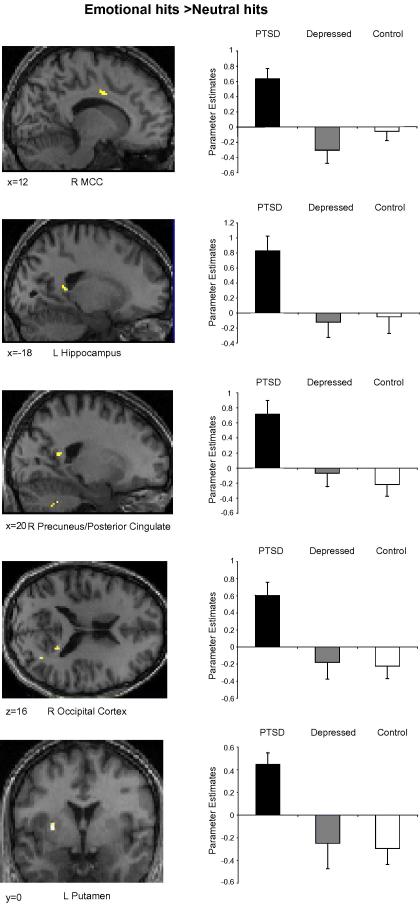
Parameter estimates (arbitrary units) for regions demonstrating additional activity in the PTSD group relative to the control and depressed groups for the emotional hit > neutral hit contrast. Statistical parametric maps thresholded at *p* < .001 uncorrected.

For the emotional > neutral hit contrast the PTSD group demonstrated greater activity than the depressed or control groups in areas of the left putamen and hippocampus, right precuneus, mid-cingulate cortex and occipital cortex (see Fig. 5 and Table 4).

For the negative > neutral hit contrast the PTSD group demonstrated greater activity than the depressed or control groups in areas of the left superior frontal cortex and right precuneus. An area of the left superior temporal gyrus was significantly less active in the PTSD group than in the depressed or control group (Table 4).

For the positive > neutral hit contrast the PTSD group demonstrated greater activity than the depressed or control groups in areas of bilateral precentral gyrus, insula, right middle temporal, right MCC, right occipital and right cerebellum (Table 4).

## 4 Discussion

In the present experiment, we investigated neural changes associated with PTSD by examining brain activity associated with memory retrieval for neutral items encoded in emotional contexts. The modulation of retrieval-related neural activity by incidental retrieval of emotional vs. non-emotional contexts is reflected in consistent patterns of activity as described in prior studies (Maratos & Rugg, 2001; Maratos et al., 2001; Smith et al., 2004, 2005). By testing patients suffering from PTSD or depression, and a trauma-exposed control group, we were able to control for non-specific effects of depression and trauma exposure, and to focus on differences specific to PTSD. Among the limitations of the study it should be noted that length of time since the index trauma was longer in the PTSD than in the trauma control group. This was a potential confound, but time since trauma is not known to have any specific effects on variables of interest to us. Here, we describe evidence for activation of common circuits in control participants and patients suffering from PTSD and depression, and also describe patterns of activation specific to the PTSD group when retrieving information encoded in emotional contexts.

### 4.1 Behavioural performance

During the encoding phase the control and PTSD groups categorised the emotional background images largely consistently with the IAPS norms. The depressed group exhibited a preference for categorising significantly more images as neutral than did the other groups. Similar evidence of reduced emotional reactivity to standard valenced stimuli (as opposed to idiographic stimuli) has been found by Rottenberg, Gross, and Gotlib (2005), and is consistent with a broad range of findings using self-report and physiological measures (Rottenberg & Gotlib, 2004). At test the PTSD group exhibited poorer recognition performance relative to the control group, in line with previous results demonstrating memory deficits in PTSD for non-trauma related stimuli (Brewin et al., 2007). However, the similar decrement observed in depressed patients mitigates against a PTSD-specific explanation. This result stands in contrast to previous neuroimaging studies of memory in PTSD (Bremner et al., 2003; Shin et al., 2004) which did not find group differences in memory performance. However, both previous studies used cued recall paradigms and both used significantly fewer stimuli than the present study, and therefore had lower statistical power to detect these effects.

### 4.2 fMRI data

#### 4.2.1 Memory retrieval effects

In order to assess the neural correlates of successful recognition we contrasted neural activity associated with correctly recognised study items with activity associated with correct rejection of new items (old > new contrast). ’Old/new’ effects common to all three groups were observed in the left middle temporal gyrus, left parietal cortex, bilateral anterior, mid, and posterior cingulate, left superior medial gyrus (dorsal medial prefrontal cortex) and bilateral prefrontal cortex. Activity in these regions has previously been reported in event-related studies of recognition memory (e.g. Henson et al., 1999; Smith et al., 2004, 2005 see Rugg et al., 2002 for a review). An additional region of interest analysis, not reported here, based on coordinates reported in Smith et al. (2004) for the same contrast revealed almost complete overlap with their findings (data available upon request).

Participants with PTSD exhibited old/new effects that differed from those of the control and depressed groups in two key regions, showing relatively increased activation in the left dorsal amygdala (close to ventral striatum) and the right middle occipital cortex. Amygdala hypersensitivity has been a consistent finding across a number of PTSD studies using a variety of methodologies and has been associated with symptom severity (Protopopescu et al., 2005; Rauch et al., 2000; Shin et al., 2004). It has been previously proposed (Phelps et al., 2001; Pitkänen, Savander, & LeDoux, 1997; Smith et al., 2004) that the amygdala responds to emotional stimuli from both internal and external sources, and specifically that the retrieval of emotional memories activates the amygdale by virtue of processing the products of retrieval where these are emotionally valenced (Smith, Stephan, Rugg, & Dolan, 2006; Smith et al., 2005). In the context of the present experiment, it may be that the proposed amygdale ‘hypersensitivity’ in PTSD patients results in arousal responses to online processing of retrieved material even when it has limited emotional content. Alternatively, the ventral striatum is associated with motivational aspects of emotionally significant stimuli (Cardinal, Parkinson, Hall, & Everitt, 2002) and is thought to represent a ‘limbic-motor interface’ where motivationally significant information can guide behaviour (Mogenson, Jones, & Yim, 1980). Additional activity here could indicate that stronger associative links between the initially neutral and paired affective stimuli were made in the PTSD group. Subdivisions between these regions may in any case be overstated for tasks like the present one. Gray (1999) notes that the combined operation of the ventral striatum and extended amygdala is essential for the association of motivational or emotion-evoking properties to previously neutral stimuli.

The PTSD group also exhibited relatively greater old/new activity in the right lateral occipital cortex. This region is known to play an important role in human object recognition (see Grill-Spector, Kourtzi, & Kanwisher, 2001) and studies in primates have demonstrated extensive connection between the amygdala and visual cortex (Amaral, Price, Pitkänen, & Carmichael, 1992). Hendler et al. (2003) presented trauma-related and neutral images to soldiers with PTSD. They found increased activity in bilateral lateral occipital complex in response to combat images presented below visual threshold. Since sensory cortex is activated to a greater degree by emotional material (Morris, Öhman, & Dolan, 1998) additional activity here in the PTSD group may reflect additional pre-attentive processing of the stimuli, perhaps mediated by feedback from the amygdala (Vuilleumier, Richardson, Armony, Driver, & Dolan, 2004). Finally, participants with PTSD exhibited relatively attenuated old/new effects in the right lateral prefrontal cortex. A number of previous studies have identified similar decreases in participants with PTSD during symptom provocation studies (Lanius et al., 2002; Shin et al., 1999) although deficits in PTSD are more reliably found in medial rather than lateral prefrontal cortex (Milad, Rauch, Pitman, & Quirk, 2006). These lateral prefrontal effects may be attributable to a decreased focus on the external physical world in favour of internal mental events (Lieberman, 2007).

#### 4.2.2 Emotion effects

In order to examine how emotion modulated the neural correlates of successful recognition we contrasted neural activity elicited by correctly recognised objects studied in emotional versus neutral contexts. In contrast to the old/new effects discussed above, however, emotion effects common to all three groups replicated findings from previous research with non-clinical participants to only a very limited extent (Maratos & Rugg, 2001; Maratos et al., 2001; Smith et al., 2004, 2005). Specifically, our analysis revealed common effects in only the pregenual and mid-cingulate cortices. Activity in the pregenual anterior cingulate was driven by retrieval of items encoded in positive relative to neutral contexts, a finding consistent with previous reviews demonstrating processing of positive emotion in this region (Vogt, 2005). Retrieval of items encoded in negative relative to neutral contexts led to increased activity in the left dorsolateral prefrontal cortex (DLPFC), a region known to be sensitive to unpleasant emotion (Gray, Braver, & Raichle, 2002). To control for item saliency we directly compared activity associated with items encoded in positive vs. negative contexts. Common effects were observed in a network of regions associated with affective processing including the posterior cingulate cortex and retrosplenial cortex, and the insula and putamen (Calder, Keane, Manes, Antoun, & Young, 2000).

There may be a number of reasons why the present study did not replicate previous effects reported in normal subjects. First, there was poorer recognition performance across all three groups, but particularly the PTSD and depressed groups, than in the previously described studies. This implies that a larger number of correct trials were likely to be categorised on the basis of guessing rather than accurate memory, which makes it difficult to detect differences between conditions. Second, the depressed patient group showed a bias for categorising contexts as neutral, presumably due to blunting of their emotional responses. This may confound our ability to detect differences between emotional and neutral conditions, especially across groups if there are differences in the way that subjects in each group process the information. Third, as already discussed, the PTSD group appear to show relatively enhanced amygdala/striatal responses to the incidental retrieval of neutral as well as emotional information. All these three factors may dilute any differences in the trials categorised as emotional or neutral retrieval, impairing our ability to detect those which may exist.

Turning to the between-group contrasts, the PTSD group demonstrated relatively greater emotion effects in several regions. When retrieving items encoded in emotional relative to neutral contexts the PTSD group recruited regions including the insula, precuneus, cingulate, hippocampus and putamen. Some of these are regions involved in the on-line processing of affective information, and we hypothesise that they act to modulate activity in the episodic retrieval system. Activity in the precuneus has been linked to mental imagery recall (Fletcher et al., 1995; Shallice et al., 1994) and could represent stronger subjective re-experiencing of emotional images (Cavanna & Trimble, 2006). In recognition memory paradigms the precuneus has been associated with enhanced familiarity for items (Yonelinas et al., 2005)—the emotional context in this case would act to increase the salience of the associated item, in turn increasing the likelihood of contextual associations being regenerated (Lundstrom, Ingvar, & Petersson, 2005; Lundstrom et al., 2003).

Effects specific to PTSD were also observed when positive hits were contrasted with neutral hits. Similarly increased activity in right precentral and right middle temporal regions has previously been reported in a study which showed positive emotional film clips to patients with PTSD (Jatzko, Schmitt, Demirakca, Weimer, & Braus, 2006). Given the relative absence of positive stimuli from investigations of PTSD (excepting Rauch et al., 2000) more research is warranted to investigate whether PTSD is associated with alterations in the processing of positive emotions.

It is interesting to note that we only observed additional amygdala/ventral striatal activity in the PTSD group for the general old > new contrast rather than the more specific emotional hit contrasts. This could indicate more general emotional arousal or motivational significance in the PTSD group when successfully retrieving non-traumatic episodic information, but not specifically associated with retrieval of items of a particular valence class.

### 4.3 Summary and general discussion

When trauma-exposed participants, or patients suffering from PTSD or depression, successfully completed a memory retrieval task common activations were evident in a predominantly left-sided network of regions previously associated with episodic memory retrieval. The results using this paradigm are consistent with a substantially intact memory retrieval network for neutral and emotionally valenced information in participants with PTSD. Relative to the control and depressed groups the PTSD group exhibited relatively greater sensitivity in networks supporting stimulus identification and episodic retrieval. While retrieving memories of all valences, enhanced activity was observed in the left amygdala/ventral striatum and right occipital cortex, regions previously found to be additionally sensitive to emotional information in PTSD (Hendler et al., 2003; Rauch et al., 2000). This may indicate that PTSD patients have enhanced sensitivity to a task in which they can anticipate they will encounter emotionally valenced stimuli. In other words, the sensitivity may be driven not so much by the retrieval of specific emotional images as by the general emotional context of the task. Other interpretations and explanations for amygdala involvement are possible, however.

Although activity common to all three groups underlying the retrieval of information encoded in emotional relative to neutral contexts was observed in anatomically appropriate regions of the cingulate (Vogt, 2005), as well as in regions of the insula and putamen, such activity was less obviously consistent with previous research using this paradigm. PTSD patients demonstrated some limited areas of increased activation in response to emotional versus neutral materials, typically confined to areas already identified with emotional processing.

There are several plausible reasons for this pattern of results. First, it is more difficult to show common effects in a three-group design, and particularly in one that uses control groups with very different types of participant. Against this, common effects were demonstrated successfully for episodic retrieval. Second, in order to ensure that stimuli used in the present study would not hold any personal relevance for our participants we had to remove some of the most upsetting images used in previous studies, thereby decreasing the aversiveness of our negative image set. Thus, our findings may be attributable to lack of variance in emotionality of the stimuli. Against this, Smith et al. (2005) reported that neural responses distinguishing emotional versus neutral materials were not dependent on the most extreme emotional stimuli.

Third, unlike many previous studies of memory in PTSD, we used visual rather than verbal stimuli. Although participants may have used verbal processing to some degree in forming the initial connections between stimuli and backgrounds, we assume the findings are more likely to reflect visual processing. Anecdotally, participants often remarked that presentation of neutral stimuli during the test phase led to the conscious retrieval of the original pictorial background. In previous research (Maratos et al., 2001; Smith et al., 2004) the principal effects of emotion in modifying retrieval processing appear to have been similar across verbal and non-verbal modalities.

Fourth, unlike in previous studies using this paradigm, all our participants had been exposed to extremely stressful events. We speculate that real-life experiences of this kind alter the appraisal of and consequent response to material presumed to be emotional taken from standard stimulus sets. For example, in the context of actual exposure to a terrorist bombing, presenting a picture of someone with a bloody face may activate a greater range of emotions, and may prompt spontaneous upward (this looks much worse) or downward (this doesn’t look so bad) comparison with the participant’s own experience. These idiosyncratic changes, perhaps involving the admixture of a greater range of different emotions, may make it particularly hard to demonstrate common or diagnosis-specific effects. If true, this interpretation has important implications for the use of such stimuli with clinical samples.

Overall, this study is consistent with the conclusion that in PTSD the networks involved in general episodic memory retrieval are largely intact, although they may demonstrate relatively greater sensitivity when the task involves emotional material. We found little evidence for distinct patterns of neural response to emotionally valenced material that was of no specific personal relevance, and it is likely that reactions to such material become more idiosyncratic. These results also rule out an important potential confound in previous studies of emotional memory in PTSD using script-driven imagery of personal traumas. Based upon the results presented here, we can conclude that the previous findings are unlikely to be due to a general disturbance in episodic retrieval of valenced information, but can be more plausibly attributed to a specific response to the presentation or retrieval of traumatic material.

## Figures and Tables

**Table 1:** Demographics and psychological questionnaire breakdown (standard deviations in parentheses)

	PTSD	Depressed	Control	
Age	36.8 (7.6)	33.6 (6.9)	32.5 (7.8)	*F*(2,45) = 1.37, *p* = .263
Sex	10 F,6 M	12 F,4 M	10 F,6 M	*x*^2^(2, *N* = 48) = 0.75, *p* = .687
Age left full time education	18.56 (3.89)	19.8 (4.02)	20.37 (4.13)	*F*(2,45) = 0.853, *p* = .433
Years since index trauma	15.65 (12.35)		4.69 (4.59)	*t*(10.06) = 3.325, *p* = .004^∗^
BAI	36.2 (12.8)	19.9 (9.0)	5.8 (5.0)	*F*(2,45) = 40.70, *p* < .001^∗^
BDI	35.3 (10.1)	30.3 (8.7)	4.7 (4.9)	*F*(2,45) = 63.16, *p* < .001^∗^
IES	79.6 (10.7)		20.3 (17.5)	*t*(24.8) = 11.53, *p* < .001^∗^
PDS	42.1 (4.3)		8.0 (5.8)	*t*(30) = 18.79, *p* < .001^∗^
CADSS	19.1 (15.4)	15.5 (14.6)	8.3 (7.7)	F(2,45) = 2.91, *p* = .065

BAI = Beck Anxiety Inventory, BDI = Beck Depression Inventory, IES = Impact of Event Scale, PDS = Post-traumatic Stress Diagnostic Scale, CADSS = Clinician Administered Dissociative States Scale.

**Table 2:** Number of items participants in each group classified as negative, neutral, or positive during Study phase, corresponding hit rates, and correct rejection rate (standard deviations in parentheses)

		PTSD	Depressed	Control
Number of items classified	Negative	50.87 (8.27)	41.56 (15.18)	50.06 (3.21)
	Neutral	46.68 (11.16)	67.68 (20.18)	50.81 (6.40)
	Positive	52.00 (6.98)	40.68 (15.42)	49.12 (5.40)
				
Hit rate	Negative	0.646 (0.163)	0.608 (0.131)	0.753 (0.137)
	Neutral	0.635 (0.140)	0.607 (0.124)	0.762 (0.120)
	Positive	0.688 (0.170)	0.584 (0.123)	0.782 (0.101)
				
Correct rejections		0.796 (0.137)	0.790 (0.085)	0.832 (0.083)

**Table 3:** Principal regions showing significant activity common to all three groups (PTSD, Depressed, Control) for the contrasts of emotional > neutral hits, negative > neutral hits, and positive > neutral hits

Region	Cluster size	MNI Coordinates (*x*, *y*, *z*)
Emotional hits > Neutral hits		
L pACC	35	−14 46 4
R aMCC	12	8 28 20
		
Negative hit > Neutral hit		
L DLPFC	28	−38 30 28
L DLPFC	6	−30 24 40
		
Positive hit > Neutral hit		
L pACC	87	−12 48 2
		
Negative hit > Positive hit		
R PCC	14	16 −12 38
L Middle Frontal	11	−30 26 42
L Occipital	11	−22 −82 −4
R Inferior Parietal	5	42 −54 38
		
Positive hit > Negative hit		
L Insula	65	−40 8 8
L Putamen		−30 6 10
L Insula	19	−32 10 −12
L Retrosplenial Cortex	10	−14 −50 14

Statistical threshold, *p* < .001 uncorrected. Each contrast exclusively masked with its cross-group interaction effects at *p* < .05 uncorrected. pACC, pregenual anterior cingulate cortex. aMCC, anterior midcingulate cortex. DLPFC, dorsolateral prefrontal cortex. PCC, posterior cingulate cortex.

**Table 4:** Regions demonstrating significantly greater (*or significantly less*) activity in the PTSD group than in the Depressed and Control groups for the Old vs. New Emotional vs. Neutral, Negative vs. Neutral, and Positive vs. Neutral contrasts

Region	Cluster size	MNI coordinates (*x*, *y*, *z*)
*Old vs. New*		
L Dorsal Amygdala/Ventral Striatum	14	−18 0 −12
R Middle Occipital	8	42 −74 6
R Middle Occipital	6	38 −82 0
(*R Lateral Prefrontal Cortex BA 46*	*11*	*40 38 10*)
		
*Emotional vs. Neutral*		
R MCC	20	14 −4 36
R Precuneus/Posterior Cingulate	8	20 −50 14
R Occipital Cortex	6	30 −68 16
L Putamen	6	−30 0 2
L Hippocampus	5	−18 −44 10
		
*Negative hits vs. Neutral hits*		
R Precuneus	16	20 −50 12
L Precentral Gyrus	22	−26 8 40
L Superior Frontal Gyrus	18	−22 6 60
(*L Superior Temporal Gyrus*	*6*	*−60 −4 −4*)
		
*Positive hits vs. Neutral hits*		
R Middle Temporal	5	44 −64 4
R MCC	5	14 −6 38
R Insula	5	44 0 4
L Precentral Gyrus	9	−26 −16 54
R Precentral Gyrus	7	58 4 26
L Insula	22	−38 −18 20
L Insula	7	−40 −8 −8
R Occipital	8	30 −70 16
L Insula	5	−44 −14 20
L Red Nucleus	19	−12 −14 −8
		
*Positive hits vs. Negative hits*		
L Superior Temporal Gyrus	13	−64 −36 22
(*L Occipital Cortex*	*10*	*−20 −64 20*)

(Inclusive masks of PTSD > control and PTSD > depressed, both at *p* < .005, uncorrected). pMCC, posterior midcingulate cortex. MCC, midcingulate cortex.
